# Granulin loss of function in human mature brain organoids implicates astrocytes in TDP-43 pathology

**DOI:** 10.1016/j.stemcr.2023.01.012

**Published:** 2023-02-23

**Authors:** Martina de Majo, Mark Koontz, Elise Marsan, Nir Salinas, Arren Ramsey, Yien-Ming Kuo, Kyounghee Seo, Huinan Li, Nina Dräger, Kun Leng, Santiago L. Gonzales, Michael Kurnellas, Yuichiro Miyaoka, Joseph R. Klim, Martin Kampmann, Michael E. Ward, Eric J. Huang, Erik M. Ullian

**Affiliations:** 1 Department of Ophthalmology, University of California San Francisco, San Francisco, CA 94143, USA; 2 Synapticure Inc, Chicago, IL 60612, USA; 3 Department of Pathology, University of California San Francisco, San Francisco, CA 94143, USA; 4 Institute for Neurodegenerative Diseases, University of California, San Francisco, San Francisco, CA, USA; 5 Medical Scientist Training Program, University of California, San Francisco, San Francisco, CA, USA; 6 Alector, Inc, South San Francisco, CA 94080, USA; 7 Regenerative Medicine Project, Tokyo Metropolitan Institute of Medical Science, 2-1-6 Kamikitazawa, Setagaya, Tokyo 156-8506, Japan; 8 Gladstone Institutes, San Francisco, CA 94158, USA; 9 Department of Stem Cell and Regenerative Biology, Harvard University, Cambridge, MA 02138, USA; 10 Stanley Center for Psychiatric Research, Broad Institute of MIT and Harvard, Cambridge, MA 02142, USA; 11 Harvard Stem Cell Institute, Harvard University, Cambridge, MA 02138, USA; 12 Department of Biochemistry and Biophysics, University of California, San Francisco, San Francisco, CA, USA; 13 National Institute of Neurological Disorders and Stroke, National Institutes of Health, Bethesda, MD, USA; 14 Neuroscience Graduate Program, University of California San Francisco, San Francisco, CA 94158, USA

**Keywords:** neurodegeneration, astrocytes, neurons, strocyte-neuronal signaling, FTD, ALS, iPSC

## Abstract

Loss of function (LoF) of TAR-DNA binding protein 43 (TDP-43) and mis-localization, together with TDP-43-positive and hyperphosphorylated inclusions, are found in post-mortem tissue of amyotrophic lateral sclerosis (ALS) and frontotemporal dementia (FTD) patients, including those carrying LoF variants in the progranulin gene (*GRN*). Modeling TDP-43 pathology has been challenging *in vivo* and *in vitro*. We present a three-dimensional induced pluripotent stem cell (iPSC)-derived paradigm—mature brain organoids (mbOrg)—composed of cortical-like-astrocytes (iA) and neurons. When devoid of *GRN*, mbOrgs spontaneously recapitulate TDP-43 mis-localization, hyperphosphorylation, and LoF phenotypes. Mixing and matching genotypes in mbOrgs showed that *GRN*^−/−^ iA are drivers for TDP-43 pathology. Finally, we rescued TDP-43 LoF by adding exogenous progranulin, demonstrating a link between TDP-43 LoF and progranulin expression. In conclusion, we present an iPSC-derived platform that shows striking features of human TDP-43 proteinopathy and provides a tool for the mechanistic modeling of TDP-43 pathology and patient-tailored therapeutic screening for FTD and ALS.

## Highlights

• *GRN*^*−/−*^ mbOrgs made of mature astrocytes and neurons show traits of TDP-43 pathology
• *GRN*^*−/−*^ astrocytes are necessary and sufficient for STMN2 mis-splicing in mbOrg
• STMN2 mis-splicing is rescued, adding progranulin to neuron and astrocyte co-cultures

## Graphical abstract

**Figure:**
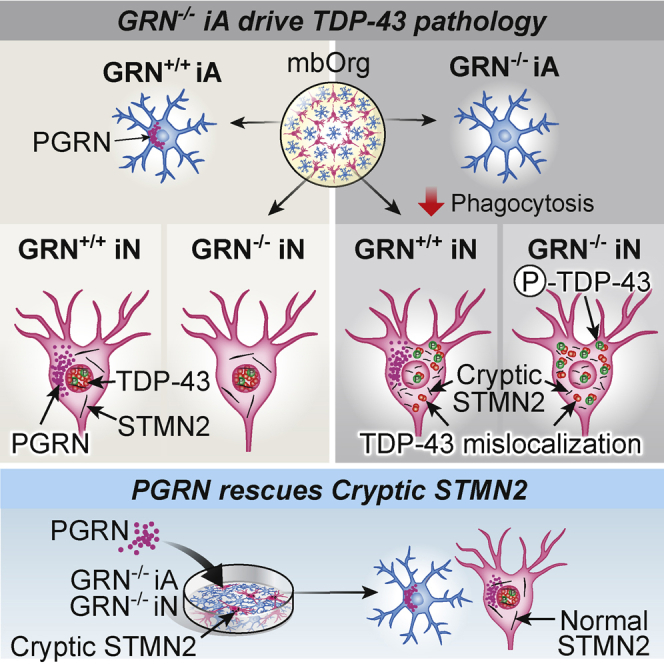

## Teaser

In this article, de Majo et al. present a novel 3D iPSC-derived model to study neurodegenerative disorders such as ALS and FTD. When devoid of *GRN* expression, these cultures present features of ALS- and FTD-associated pathology hardly ever observed *in vitro*. These phenotypes are shown to be primarily driven by diseased astrocytes and can be rescued by pro-granulin supplementation.

## Introduction

Frontotemporal dementia (FTD) and amyotrophic lateral sclerosis (ALS) are two fatal neurodegenerative diseases. ALS is estimated to affect 2.1 cases per 100,000 people per year (Chiò et al., 2013), while FTD is the second most common cause of dementia for people under 65 years of age (Knopman and Roberts, 2011). ALS and FTD are now thought to be different manifestations of the same disease spectrum, with ALS primarily affecting the motor system and FTD presenting with a variety of symptoms affecting behavioral, executive, language, and motor functions. The two clinical entities can also occur in the same patients, with approximately 10%–15% of ALS patients diagnosed with FTD features (FTD-ALS) and approximately 50% of ALS patients developing some cognitive impairment (Burrell et al., 2016). TAR DNA binding protein (*TARDBP*) gene encodes for the TDP-43 protein, which plays a pivotal role in these two devastating neurodegenerative disorders. TDP-43 was also recently implicated in limbic-predominant age-related TDP-43 encephalopathy (LATE) (Nelson et al., 2022). Although TDP-43 is an RNA/DNA binding protein that physiologically resides in the nucleus, hyperphosphorylated extranuclear inclusions are found in neuronal cells of approximately 45% of patients with FTD and approximately 97% of patients with ALS (Ling et al., 2013; Neumann et al., 2006). Loss of nuclear TDP-43 results in defective splicing of several transcripts, among which the most thoroughly described is the cryptic splicing of Stathmin 2 (*STMN2*), a neuron-specific gene important for neuronal survival (Akiyama et al., 2022; Klim et al., 2019; Melamed et al., 2019; Prudencio et al., 2020).

Approximately 5%–10% of all FTD patients harbor mutations in the granulin (*GRN*) gene. (Baker et al., 2006; Cruts et al., 2006; Seelaar et al., 2008). *GRN* transcripts are composed of 13 exons and encode for the progranulin (PGRN) protein, a highly conserved, approximately 80-kDa protein involved in lysosomal function, neuronal survival, and inflammation (Rhinn et al., 2022). The vast majority of the *GRN* mutations are dominant heterozygous loss-of-function variants that lead to haploinsufficiency with a consequent lower expression of PGRN. These patients have been reported to harbor TDP-43 proteinopathy in their frontal and/or temporal lobes at post-mortem examination (Baker et al., 2006; Mackenzie, 2007). The *GRN* gene has been further linked to Alzheimer’s disease and LATE, suggesting that this gene plays roles in multiple neurodegenerative diseases (Bellenguez et al., 2020; Nelson et al., 2019; Viswanathan et al., 2009)

To date, modeling TDP-43 proteinopathy *in vitro* or *in vivo* has been a challenge, with most *in vitro* models applying exogenous stress to replicate pathology akin to what is observed in post-mortem ALS/FTD central nervous system (CNS) tissue. Within these paradigms, induced pluripotent stem cells (iPSCs) hold great promise; they allow for patient- and tissue-specific human-derived *in vitro* models, which can be used for therapeutic screening and phenotype testing (Palomo et al., 2019). As an example, recent work has established the usefulness of iPSC models for the study of *GRN* loss of function (LoF) (Lee and Huang, 2017; Rosen et al., 2011). *GRN* depletion in human iPSC-derived neurons causes cell autonomous changes in signaling (Almeida et al., 2012) and cellular stress (Rosen et al., 2011). Interestingly, these *in vitro* studies did not replicate the characteristic TDP-43 pathology observed in patients (Ahmed et al., 2010; Ward et al., 2014). One possible explanation for this is the fact that *GRN* is also expressed in glia, such as microglia and astrocytes, raising the likelihood that *GRN* LoF causes pathology in a non-cell autonomous manner (Kelley et al., 2018; Zhang et al., 2014; 2016). Recent studies have further implicated glial cells, including astrocytes and microglia, in neurodegenerative disease progression (Desai et al., 2010; Hansen et al., 2018; Verkhratsky et al., 2010). Indeed, several lines of evidence from *GRN* knockout mouse models indicate PGRN deficiency induces glial complement activation and non-autonomous microglia-mediated synaptic pruning that subsequently leads to neurodegeneration (Lui et al., 2016). The role of astrocytes, however, has been less thoroughly characterized and important questions remain, such as whether disease-associated astrocytes are capable of inducing TDP-43 pathology and whether human models of FTD can show pathology similar to that found in post-mortem human brain.

To address this challenge, we applied our previously developed iPSC-derived 3D co-culture model (Krencik et al., 2017; Liu et al., 2020) composed of mature cortical-like neurons and astrocytes, assembled in precise ratios and numbers, to study *GRN* LoF in FTD. When devoid of granulin expression (*GRN*^−/−^), our model develops features of TDP-43 pathology, including cryptic *STMN2* (CrSTMN2) splicing, and extranuclear and hyperphosphorylated TDP-43 inclusions. This study presents the first *in vitro* model showing robust evidence of numerous FTD/ALS pathology markers spontaneously developing, overcoming the need of exogeneous chemical-induced stress or overexpression. Furthermore, we obtained partial phenotype rescue when *GRN*^−/−^ cells were treated with exogenous full-length PGRN, demonstrating that the development of TDP-43 pathology depends on PGRN expression. We believe this model could provide insight into cell biological mechanisms, leading to TDP-43 pathology and offer a platform for patient-tailored phenotype and therapeutic screening for FTD and ALS patients with suspected or confirmed TDP-43 proteinopathy.

## Results

**Figure 1:**
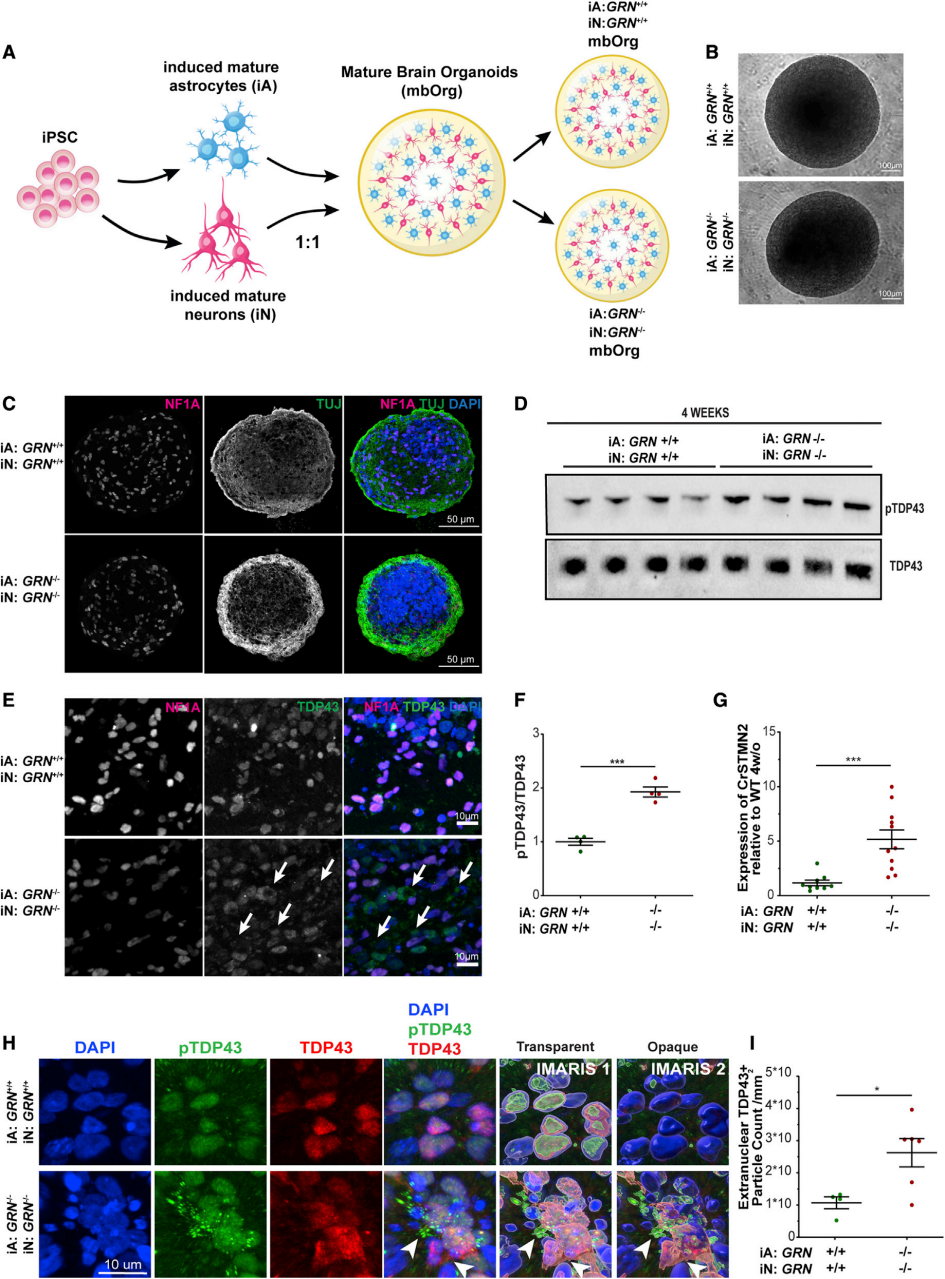
GRN^−/−^ mbOrgs show features of human TDP-43 proteinopathy after 4 weeks in culture (A) Diagram showing the process by which mbOrgs are differentiated and assembled. Briefly, iPSCs are differentiated into mature cortical-like astrocytes and neurons and assembled in a 1:1 ratio in 3D co-cultures called mbOrg. The mbOrg are grown for four weeks and then analyzed. (B) Brightfield image of two mbOrgs (*GRN*^*+/+*^, *GRN*^*−/−*^) kept in culture for 4 weeks (scale bar, 100 μm). (C) Confocal images of mbOrg slices (*GRN*^*+/+*^ or *GRN*^*−/−*^) showing expression of NF1A, TUJ, and DAPI after 4 weeks in culture (scale bar, 50 μm). (D) Western blot of mbOrgs whole lysate showing higher expression of pTDP-43 in *GRN*^*−/−*^ compared with *GRN*^*+/+*^ mbOrgs when normalized to total TDP-43. (F) Western blot quantification showing significantly higher expression of pTDP-43 in *GRN*^*−/−*^ compared with *GRN*^*+/+*^ mbOrgs when normalized to total TDP-43 (n = 4, unpaired t test, two tailed, p < 0.001, each n represents approximately 50 mbOrgs and was repeated independently four times). (E) Confocal images of mbOrg slices (*GRN*^*+/+*^ or *GRN*^*−/−*^) showing expression of NFIA, TDP-43 and DAPIafter 4 weeks in culture (scale bar, 10 μm). (G) Quantification of CrSTMN2 expression using qPCR showing significantly higher expression in *GRN*^*−/−*^ compared to *GRN*^*+/+*^ mbOrgs (n = 11±, unpaired t test, two tailed, p ≤ 0.001, each n represents approximately 50 mbOrgs and the experiment was repeated independently four times). (H) IMARIS 3D reconstruction of TDP-43 and pTDP-43 staining in 4-week-old mbOrgs (scale bar, 10 μm). (I) quantification of extranuclear TDP-43 particle count per square millimeter, showing significantly higher count in *GRN*^*−/−*^ compared with *GRN*^*+/+*^ mbOrgs. Each dot represents one independent mbOrg (*GRN*^*+/+*^ n = 4, *GRN*^*−/−*^ n = 6, unpaired t test, two tailed, p < 0.05, each n is one mbOrg and the experiment was repeated independently three times). For all graphs, data are presented as mean ± standard error of the mean.

To investigate the role of astrocytes in *GRN* LoF, we modified a previously described protocol to study glial-neuronal interactions (Krencik et al., 2017) (Figure 1A). This approach entails generating neurogenin 2 (NGN2)-inducible cortical-like neurons (iNeurons [iN]), which readily form synapses (Fernandopulle et al., 2018), and mature cortical-like astrocytes (iAstrocytes [iA]) (Krencik et al., 2015; Krencik and Zhang, 2011), and assembling them into three-dimensional (3D) organoid-like structures at defined numbers and ratios of neurons and astrocytes (termed mature brain organoids [mbOrgs]) (Figure 1B). This approach allows us to better model the ratio of astrocytes to neurons thought to comprise the human cortex, as well as mix and match neurons and astrocytes derived from either isogenic wild-type (*GRN*^+/+^) iPSCs or isogenic *GRN* knockout (*GRN*^−/−^) iPSCs (Figure S2G). Importantly, *GRN*^−/−^ mbOrgs show a complete loss of PGRN (Figures S1B and S1E). Using this approach, we interrogated the pathological phenotypes of *GRN*^+/+^ and *GRN*^−/−^ mbOrgs. Using transmission light microscopy and confocal imaging, we showed that mbOrgs formed from *GRN*^+/+^ or *GRN*^−/−^ iPSCs both developed into uniform spheres containing a readily detectible array of astrocytes and neurons (Figures 1B and 1C). We first looked at standard markers characteristic of iPSC-derived neurons and astrocytes, by immunostaining. Aquaporin 4 (*AQP4*), an astrocyte specific gene well expressed by astrocytes (Krencik et al., 2015), was strongly and uniformly expressed in both *GRN*^+/+^ and *GRN*^*−/−*^ mbOrgs (Figure 3A). Similarly, we found a strong and widespread expression of neuronal microtubule-associated protein 2 (MAP2) expression in both mbOrgs (Figure 3B). Both results indicate overall stable expression of these astrocyte and neuronal markers in *GRN*^+/+^, as well as *GRN*^−/−^ mbOrgs.

*GRN*-associated FTD TDP-43 proteinopathy is characterized by increased levels of phospho-TDP-43 (pTDP-43) and abnormal TDP-43 cytoplasmic accumulation, seen in postmortem CNS tissue (Baker et al., 2006; Mackenzie, 2007). To assess the TDP-43 pathology in our model, we measured the levels of both pTDP-43, using a ser409/410-specific pTDP-43 antibody, and total TDP-43 by performing western blotting in *GRN*^+/+^ and *GRN*^*−/−*^ mbOrgs lysates after 4 weeks of culture (Figure 1D). Quantification of the pTDP-43 to TDP-43 ratio revealed a clear increase in the phosphorylated form of TDP-43 in *GRN*^*−/−*^ mbOrgs (Figure 1F), similar to what has been described in post-mortem tissue (Baker et al., 2006; Mackenzie, 2007). We next performed immunostaining for TDP-43 in sections of 4-week-old mbOrgs and found clear evidence of extranuclear localization of TDP-43 in *GRN*^−/−^ mbOrgs, but not in *GRN*^+/+^ mbOrgs, where TDP-43 was mostly co-localized with nuclei (Figure 1E). IMARIS reconstruction and quantification of confocal images from TDP-43 staining demonstrated an increase in extranuclear TDP-43 particle count in *GRN*^−/−^ mbOrgs (Figures 1H and 1I). Recent work has shown that a key function of TDP-43 in healthy cells is mRNA splicing repression in the nucleus, whereas in disease TDP-43 nuclear depletion results in a number of mis-spliced transcripts (Brown et al., 2022). Among these, *STMN2* is the most thoroughly studied; mis-splicing of *STMN2* transcripts (CrSTMN2) has been considered a robust indicator of TDP-43 pathology and correlates with the level of pTDP-43 (Klim et al., 2019; Melamed et al., 2019; Prudencio et al., 2020). Thus, to determine if CrSTMN2 transcripts can be detected in our model system showing TDP-43 mis-localization, we adapted a method (Klim et al., 2019) to develop a sensitive quantitative PCR (qPCR)-based assay and found a highly significant, approximately 4-fold increase in CrSTMN2 in the *GRN*^*−/−*^ versus *GRN*^*+/+*^ mbOrgs (Figure 1G). We next asked if these unique features of human neurodegenerative disease progress over time in *GRN*^*−/−*^ mbOrgs, as would be expected if these features represent cellular mechanisms that could be relevant to human disease progression. To test this, we looked at an earlier 2-week time point and assessed the presence of these same features that are robustly present at four weeks. We found that, while there was a trend, variable results caused these features to have not yet reached statistical significance at the earlier time point, consistent with a progression of relevant cellular signaling driving these features (Figures S1A, S1C, and S1D). Taken together, these data demonstrate a remarkable degree of human-specific FTD pathological phenotypes recapitulated in the iPSC-derived mbOrg model and are, to the best of our knowledge, the first demonstration of multiple TDP-43-associated pathological phenotypes shown in an unperturbed *in vitro* model system.

**Figure 2:**
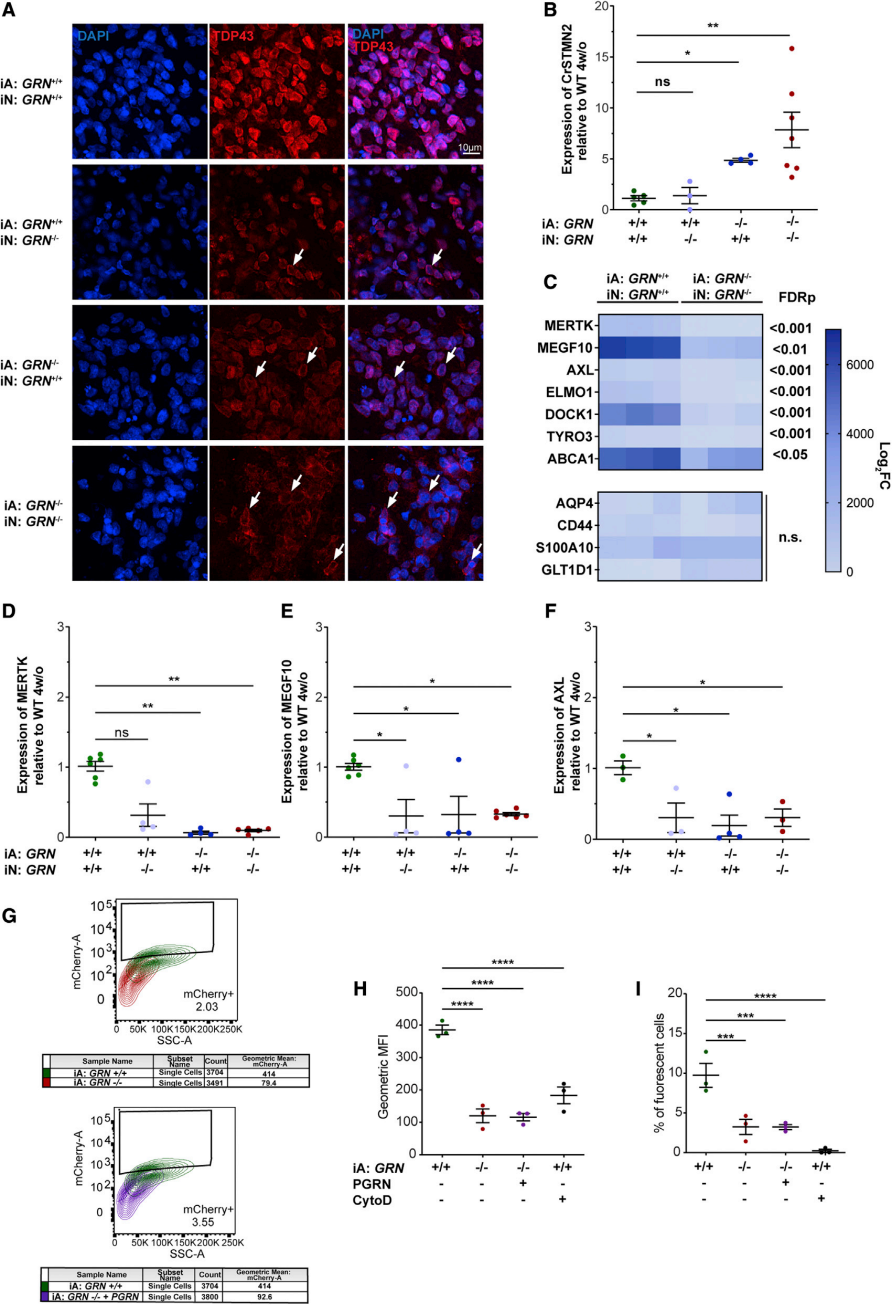
GRN^−/−^ mature astrocytes drive *STMN2* mis-splicing in mbOrgs and show evidence of defective phagocytosis (A) Qualitative immunohistochemistry of mbOrg slices (*GRN* iA: +/+ iN: +/+, *GRN* iA: +/+ iN: −/−, *GRN* iA: −/− iN: +/+, *GRN* iA: −/− iN: −/−) showing expression of TDP-43 and DAPI after four weeks in culture (scale bar, 10 μm). (B) Quantification of CrSTMN2 expression using qPCR showing increasingly significant CrSTMN2 expression in *GRN* iA: −/− iN: +/+ and *GRN* iA: −/− iN: −/− compared with *GRN* iA: +/+ iN: +/+ and *GRN* iA: +/+ iN: −/− mbOrgs (n = 4, one-way ANOVA followed by multiple comparison, ^∗^p < 0.05 ^∗∗^p < 0.005, each n represents approximately 50 mbOrgs and the experiment was repeated independently three times). (C) At the top, expression heatmap showing bulk RNA sequencing values of seven genes linked to phagocytosis in 4-week-old mbOrgs. All the genes listed here are differentially expressed in the *GRN*^*+/+*^ samples versus *GRN*^*−/−*^ samples. Specifically, all the genes listed here are less expressed in the 4-week-old *GRN*^*−/−*^ compared with the *GRN*^*+/+*^ mbOrgs (false discovery rate [FDR] <0.05, n = 3). At the bottom expression heatmap of astrocyte markers that are not differentially expressed in the 4-week-old *GRN*^*−/−*^ compared with the *GRN*^*+/+*^ mbOrgs. Fold change (FC). (D–F) qPCR analysis of three (MERTK, MEGF10, and AXL) of the seven phagocytosis markers analyzed with RNA sequencing in all four conditions: *GRN* iA: +/+ iN: +/+, *GRN* iA: +/+ iN: −/−, *GRN* iA: −/− iN: +/+, *GRN* iA: −/− iN: −/− (n = 4, one-way ANOVA followed by multiple comparison, ^∗^p < 0.05 ^∗∗^p < 0.005 each n represents approximately 50 mbOrgs and the experiment was repeated independently three times). (G) Graphic representation of the clustering analysis depicting the changes of phagocytic activity in the different conditions (iA *GRN*^*+/+*^ in green, iA *GRN*^*−/−*^ in red and iA *GRN*^*−/−*^ + PGRN in purple). Cells were treated with mCherry labeled rat synaptosome and analyzed at the cytofluorimeter as described in Dräger et al., 2022. (H, I) Quantification of FACS analysis showing geometric mean fluorescence intensity (MFI) and percentage of fluorescent cells phagocyted by the mature astrocytes. iA *GRN*^*−/−*^ and iA *GRN*^*−/−*^ + PGRN phagocyte significantly lower amount of mCherry-labeled synaptosome according to both parameters compared to iA *GRN*^*+/+*^ (n = 3, one-way ANOVA followed by multiple comparison, ^∗∗∗^p < 0.0005 ^∗∗∗∗^p < 0.00005 each n represents approximately 250,000 cells and the experiment was repeated independently three times). Negative control showed in black (iA *GRN*^*+/+*^ treated with cytoD). For all graphs, data are presented as mean ± standard error of the mean.

These results show that *GRN* LoF in both iPSC-derived neurons and astrocytes in our 3D platform display a remarkable array of phenotypes relevant to FTD-TDP. We next wanted to investigate if *GRN* LoF is required in both cell types or if we can detect evidence of pathology when either neurons or astrocytes are *GRN*^−/−^. To investigate this, we took advantage of the assembled nature of the mbOrgs and made heterotypic cultures containing all possible combinations of *GRN*^−/−^ or *GRN*^*+/+*^ neurons + *GRN*^*−/−*^ or *GRN*^*+/+*^ astrocytes (either *GRN*^*−/−*^ neurons + *GRN*^+/+^ astrocytes or *GRN*^+/+^ neurons *+ GRN*^*−/−*^ astrocytes, along with the control both-cell-type *GRN*^+/+^ and both-cell-type *GRN*^−/−^ mbOrgs). Immunostaining for TDP-43 showed expected pathology in the both-cell-type *GRN*^−/−^ mbOrgs. We also found TDP-43 pathology in the *GRN*^+/+^ neurons + *GRN*^−/−^ astrocytes mbOrgs (Figure 2A). To further quantitatively examine the heterotypic cultures, we assessed CrSTMN2. As expected, quantification of CrSTMN2 showed the most severe phenotype in both-cell-type *GRN*^−/−^ mbOrgs. Surprisingly, we found a robust CrSTMN2 increase in *GRN*^+/+^ neurons + *GRN*^−/−^ astrocytes mbOrgs (Figure 2B), but not in *GRN*^−/−^ neurons + *GRN*^+/+^ astrocytes mbOrgs, confirming what was observed by immunocytochemistry. These results demonstrate that *GRN*^−/−^ astrocytes are sufficient to induce robust TDP-43 and CrSTMN2 phenotypes in mbOrgs, even in the presence of *GRN*^+/+^ neurons, and indicate that the diseased human astrocytes can drive neurodegenerative phenotypes in healthy human neurons.

To further characterize the effect of *GRN* LoF on iA, we performed bulk RNA sequencing (Figures 2D and 2E) to examinee gene expression in *GRN*^+/+^ and *GRN*^−/−^ mbOrgs after 4 weeks in culture. Interestingly, we found that a set of astrocyte genes known to be involved in phagocytosis (Figure 2C upper panel), but not other astrocyte specific marker genes, were down-regulated in the *GRN*^*−/−*^ mbOrgs at 4 weeks when compared with *GRN*^+/+^ mbOrgs (Figure 2C lower panel). Confirmation with qPCR shows MER proto-oncogene, tyrosine kinase (MERTK), multiple EGF-like domains 10 (MEGF10), and AXL receptor tyrosine kinase (AXL) down-regulation in *GRN*^−/−^ mbOrgs. Interestingly, all three phagocytosis-related genes were also significantly down-regulated in mbOrgs composed of *GRN*^+/+^ neurons + *GRN*^−/−^ astrocytes. For the *GRN*^*−/−*^ neurons + *GRN*^+/+^ astrocytes mbOrgs, both MEGF10 and AXL were significantly down-regulated and there was a non-significant trend for down-regulation for MERTK (Figures 2D–2F). These results suggest that *GRN* LoF in astrocytes and/or neurons can lead to a down-regulation of phagocytosis-related astrocytes genes. To directly assess the functional consequences of *GRN* loss in astrocytes, we performed a synaptosome phagocytosis assay in *GRN*^+/+^ or *GRN*^*−/−*^ astrocytes two-dimensional (2D) cultures. The results point to a profound deficit in synaptosome phagocytosis in the *GRN*^*−/−*^ astrocytes (Figures 2G–2I). We attempted to rescue this deficit with the addition of recombinant PGRN; however, the tested conditions were not sufficient to revert the phagocytosis deficit (Figures 2G–2I). A similar treatment with PGRN in 2D astro-neuronal cultures for up to 4 weeks failed to rescue the phagocytosis-related down-regulated genes MERTK and MEGF10 (Figures 1H and 1K). Overall, these data show that *GRN* LoF leads to changes in astrocyte phagocytosis and that the genes associated with astrocyte phagocytosis are regulated both cell autonomously and non-cell autonomously.

**Figure 3:**
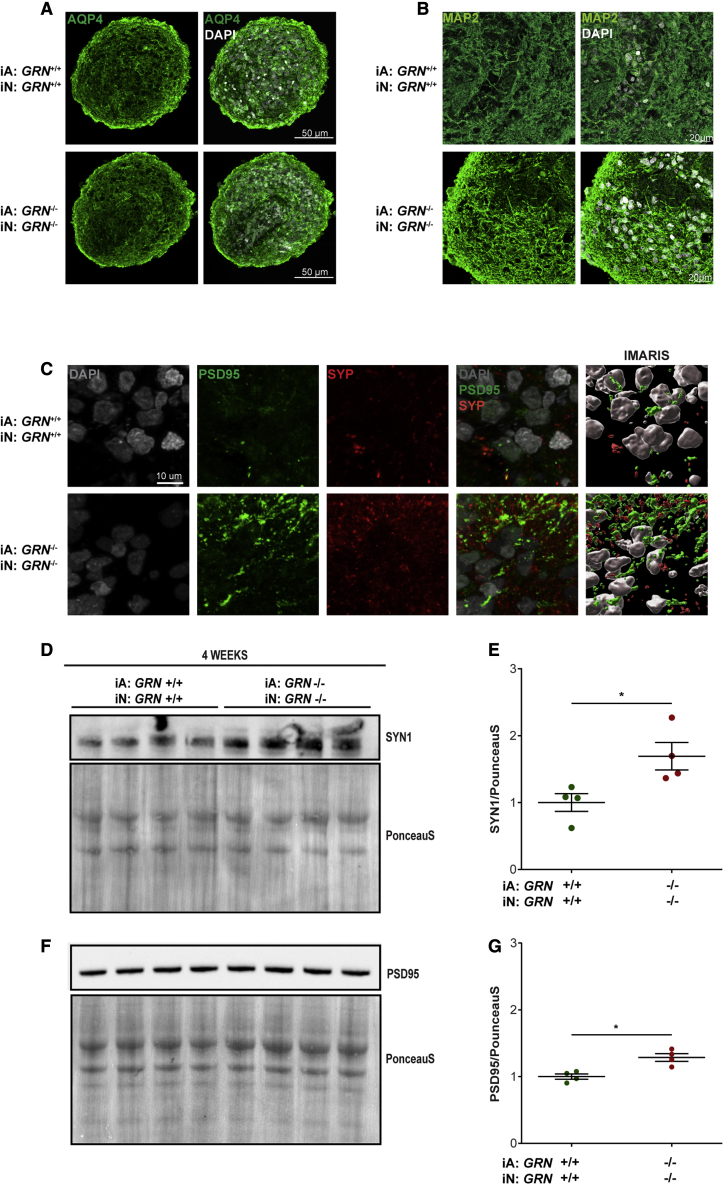
GRN^−/−^ mbOrgs show higher synaptic density when compared with GRN^+/+^ mbOrg (A) Confocal images of mbOrg slices (*GRN*^*+/+*^ or *GRN*^*−/−*^) showing evenly distributed expression of AQP4 and DAPI (scale bar, 50 μm). (B) Confocal images of mbOrg slices (*GRN*^*+/+*^ or *GRN*^*−/−*^) showing evenly distributed expression of MAP2 and DAPI (scale bar, 20 μm). (C) IMARIS 3D reconstruction of PSD95 and synaptophysin (SYP) staining in 4-week-old mbOrg (scale bar, 10 μm) showing higher synaptic density in *GRN*^*−/−*^ compared with *GRN*^*+/+*^ mbOrg. (D, E) Western blot of mbOrg whole lysate showing higher expression on Synapsin1 (SYN1) in *GRN*^*−/−*^ compared with *GRN*^*+/+*^ mbOrgs when normalized to PonceauS staining and its quantification (E) (n = 4, unpaired t test, two tailed, p < 0.05, each n represents approximately 50 mbOrgs and was repeated independently four times). (F, G) Western blot of mbOrgs whole lysate showing higher expression on PSD95 in *GRN*^*−/−*^ compared with *GRN*^*+/+*^ mbOrgs when normalized to PonceauS staining and its quantification (G) (n = 4, unpaired t test, two tailed, p < 0.05, each n represents approximately 50 mbOrgs and was repeated independently four times). For all graphs data are presented as mean ± standard error of the mean.

Previous work has uncovered a critical role for astrocyte phagocytosis in regulating synapses in various regions of the mouse CNS with specific involvement of MERTK and MEGF10 (Chung et al., 2013). We wondered whether the deficits we observed in *GRN*^*−/−*^ astrocyte phagocytosis may be associated with corresponding increases in synapses in our model. Therefore, we looked for any synaptic phenotypes associated with loss of phagocytosis in the mbOrgs. First, we immunostained mbOrgs for the presynaptic marker synaptophysin (SYP) and post-synaptic marker post-synaptic density protein 95 (PSD-95). Confocal microscopy analysis and IMARIS 3D reconstruction showed that in comparison with 4-week-old *GRN*^*+/+*^ mbOrgs. *GRN*^*−/−*^ mbOrgs contain more SYP and PSD-95 puncta (Figure 3C). We next wanted to quantitatively assess the expression levels of synaptic markers and performed western blotting for both pre- and post-synaptic markers synapsin and PSD-95, respectively. The results revealed an increase in both pre- and post-synaptic protein in the *GRN*^*−/−*^ mbOrgs (Figures 3D and 3E). Thus, *GRN* loss is associated with effects on synapses consistent with previous studies (Petoukhov et al., 2013; Tapia et al., 2011; Uesaka et al., 2018; L. Wang et al., 2022).

**Figure 4:**
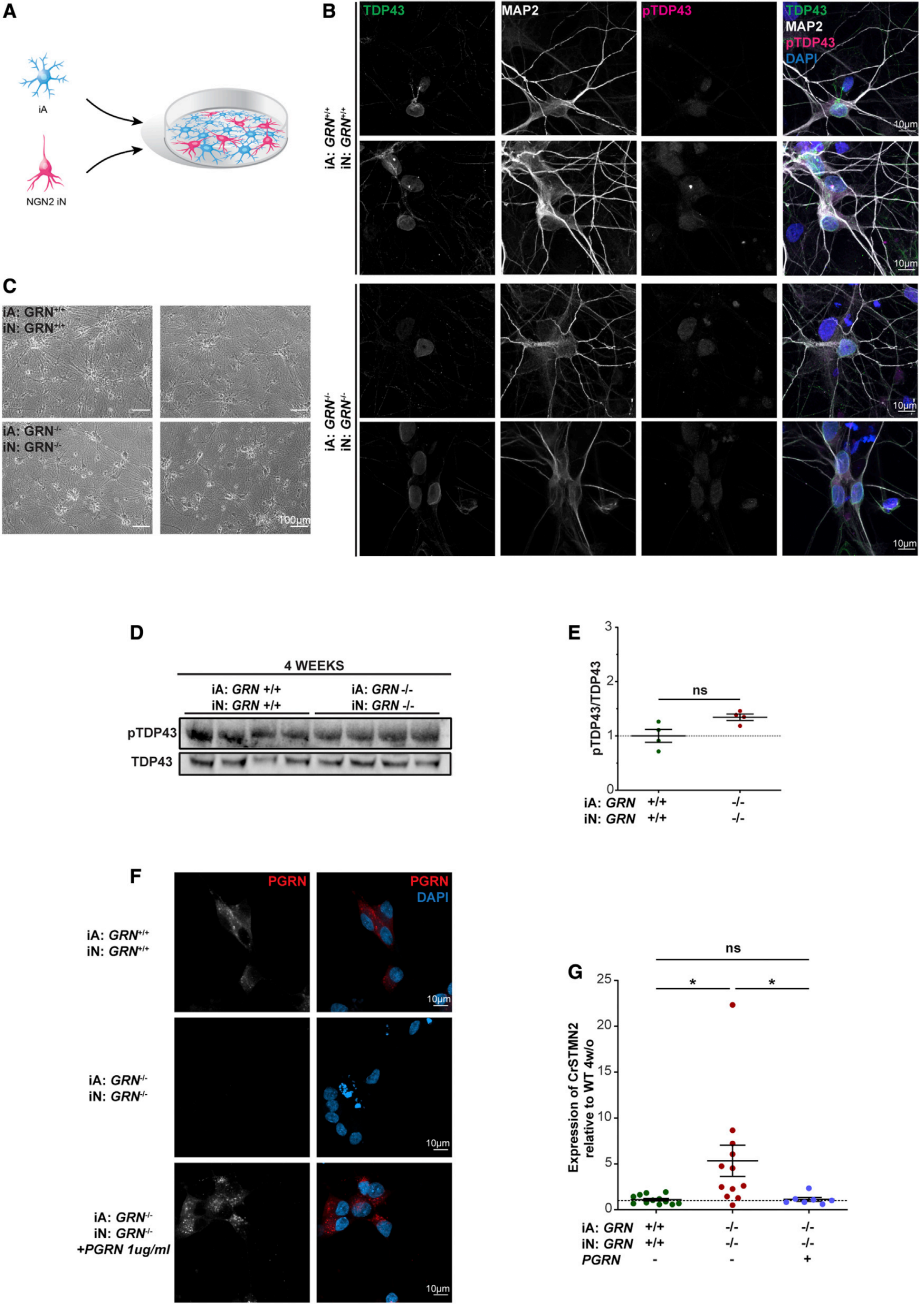
*STMN2* mis-splicing can be rescued in 2D (A) Diagram showing the process by which 2D cultures are made. Briefly, iPSCs are differentiated into mature cortical-like astrocytes and neurons and assembled in a 1:1 ratio in 2D co-cultures. The co-cultures are grown for 4 weeks and then analyzed. (B) Representative ICC images of 2D cultures stained for TDP-43, pTDP-43, MAP2, and DAPI (scale bar, 10 μm). (C) Brightfield image of 2D cultures (*GRN*^*+/+*^, *GRN*^*−/−*^) at the 4-week timepoint (scale bar, 100 μm). (D) Western blot of 2D co-cultures whole lysate of pTDP-43 and total TDP-43 in *GRN*^*+/+*^ and *GRN*^*−/−*^ 2D co-cultures. (E) Western blot quantification showing similar expression on pTDP-43 in *GRN*^*−/−*^ compared with *GRN*^*+/+*^ 2D co-cultures when normalized to total TDP-43 (n = 4, unpaired t test, two tailed, p > 0.05, each n represents approximately 1 × 10^6^ cells and was repeated independently four times). (F) Representative ICC images of 2D cultures stained for PGRN and DAPI showing positive staining in both *GRN*^*+/+*^ and PGRN treated *GRN*^*−/−*^but no PGRN staining in *GRN*^*−/−*^ co-cultures (scale bar, 10 μm). (G) Quantification of CrSTMN2 expression using qPCR showing significantly higher expression in *GRN*^*−/−*^ compared with *GRN*^*+/+*^ 2D co-cultures. The difference is rescued when *GRN*^*−/−*^ are treated with PGRN for 4 weeks (n = 10, one-way ANOVA followed by multiple comparison, ^∗^p < 0.05, ns p > 0.05, each n represents approximately 1 × 10^6^ cells and was repeated independently three times). For all graphs, data are presented as mean ± standard error of the mean.

Overall, these findings indicate that *GRN*^*−/−*^ astrocytes are likely sufficient to induce TDP-43 pathology, as well as a significant increase in CrSTMN2 in neurons in the 3D mbOrgs. We then decided to investigate whether the same two findings are present in a 2D culture system. We performed the co-culture experiments as illustrated in Figure 4A. Both *GRN*^+/+^ and *GRN*^*−/−*^ co-cultures of astrocytes and neurons look uniformly healthy at 4 weeks (28 DIV) (Figure 4B). When immunostained for MAP2, both cultures showed robust and extensive dendritic arbors (Figure 4B). As previously reported, staining for TDP-43 and pTDP-43 failed to show an overt pathological TDP-43 associated signal. Furthermore, western blot analyses for the ratio of pTDP-43 to total TDP-43 showed no significant increase at 4 weeks in *GRN*^*−/−*^ 2D co-cultures. We then assessed the expression of CrSTMN2 in the 2D co-cultures. We found a significant increase in the expression of CrSTMN2 at 4 weeks in 2D co-cultures of *GRN*^*−/−*^ astrocytes with *GRN*^*−/−*^ neurons (Figure 4G). This result led us to investigate whether this more subtle TDP-43-associated phenotype might be reversible. Although phenotypes are not as severe in the 2D co-culture, they are amenable to exogenous compound rescue experiments. As proof of principle, we tried rescuing the CrSTMN2 phenotype by treating the cells with recombinant PGRN. We first determined what, if any, concentration of PGRN shows clear cellular uptake. We determined by immunostaining that 1 μg/mL recombinant PGRN fed every 3 days for 28 days leads to a robust rescue of PGRN deficiency in *GRN*^*−/−*^ neuronal and *GRN*^*−/−*^ astrocytes cell bodies (Figure 4F). We next examined CrSTMN2 in PGRN-treated cells versus control and found a consistent and significant rescue of CrSTMN2 in 2D co-cultures of *GRN*^*−/−*^astrocytes and *GRN*^*−/−*^ neurons (Figure 4G). These are, to our knowledge, the first data demonstrating a rescue of *GRN* LoF CrSTMN2 expression increases.

## Discussion

Here, we characterize an *in vitro* human iPSC-derived neuro-glia 3D model of TDP-43 proteinopathy in a context of PGRN deficiency. While iPSC-induced neurons have provided numerous cell-autonomous biological insights into neurodegeneration, most models do not recapitulate multiple aspects of TDP-43-associated pathology. These phenotypic challenges have made therapeutic discovery difficult for TDP-43 proteinopathies (Buratti, 2020). Our focus on iPSC neuron-glial interactions have yielded a 3D paradigm spontaneously reproducing overt pathological features of TDP-43 proteinopathy. Interestingly, while TDP-43 proteinopathy was not noticeable in 2D co-culture, TDP-43 LoF characterized by *STMN2* mis-splicing was recapitulated in both 2D and 3D co-culture models.

Our approach is distinct in that we use a simple, reproducible, and straightforward engineered system to model spontaneous TDP-43 pathology. Indeed, our 3D iPSC-induced cortical-like neuron and astrocyte co-culture with *GRN* loss strikingly and consistently recapitulated human specific TDP-43 cell pathology reported in human FTD patient brain. Importantly, these phenotypes are not seen in the same individually cultured iPSC-induced neurons or astrocytes and are much milder in our 2D co-culture paradigm. Similarly, two recent studies highlighted evidence of mild TDP-43 pathology in 2D ALS-iPSC-derived neuron and 100-day-old *GRN* knockout neuronal cultures when compared with healthy controls, with the ALS study showing evidence of *STMN2* mis-splicing (Bossolasco et al., 2022; Coyne et al., 2021). The reason for difference in phenotype severity between 2D and 3D co-cultures is not entirely clear, but we have previously found that astrocytes in 3D culture are significantly more complex and resemble the highly complex morphology of human astrocytes *in vivo* (Krencik et al., 2017). Furthermore, these previously published results suggest that control iPSC-derived human astrocytes in 3D are not highly reactive by 4 weeks *in vitro* (Krencik et al., 2015), potentially providing a healthy baseline with which to compare disease-causing mutations.

The data presented here provide strong evidence that *GRN*^*−/−*^ astrocytes drive *STMN2* mis-splicing in both *GRN*^*−/−*^ and *GRN*^*+/+*^ neurons in mbOrg. Indeed, the 3D mbOrgs are advantageous in allowing easy mixing and matching of cellular genotypes with cells used to assemble the cultures. Thus, our ability to generate mixed *GRN*^*+/+*^ neurons + *GRN*^*−/−*^ astrocytes showing nearly as strong crSTMN2 and TDP-43 phenotypes of full *GRN*^*−/−*^ neuron and astrocyte cultures indicate that *GRN* LoF in human astrocytes can lead to TDP-43 LoF in neurons. This finding is mechanistically important and adds to a growing body of literature suggesting that disease-associated astrocytes and more generally glia can drive cell death and neuronal dysfunction (Huang et al., 2022; Leng et al., 2022; Liddelow et al., 2017; Taha et al., 2022; Zhang et al., 2020).

Our investigation of phagocytic activity assays in *GRN*^−/−^ iA demonstrated significant deficit when compared with *GRN*^*+/+*^ iA. Phagocytic changes have been reported in *GRN*^*−/−*^ microglia (Guan et al., 2020; Lui et al., 2016) and in microglia differentiated from ALS peripheral blood mononuclear cells (PBMCs) when compared with control PBMC-derived microglia (Quek et al., 2022). It has also been shown that diseased-induced astrocytes lose the ability to engulf synapses and show decreased expression of phagocytosis receptor *MEGF10* and *MERTK* (Liddelow et al., 2017). We show that *GRN*^−/−^ iA when cultured in 3D mbOrgs for 4 weeks also display differential expression of phagocytosis markers when compared with *GRN*^*+/+*^ iA. A significantly lower expression of *MERTK*, *MEGF10*, and *AXL* was confirmed by qPCR in *GRN*^−/−^ iA in both 3D and 2D co-cultures and was not rescued by PGRN treatment. *MEGF10* and *MERTK* have been specifically implicated in synaptic pruning and maturation in both in the developing and adult brain (Chung et al., 2013; Lee and Chung, 2019); their down-regulation could explain the significantly higher expression of pre- and post-synaptic markers observed in our 3D *GRN*^−/−^ mbOrgs when compared with *GRN*^*+/+*^*.* Overall, this model recapitulates key disease astrocyte-like features observed in neurodegenerative disorders; it is, therefore, not surprising that *GRN*^−/−^ iA are able to drive the disease phenotype in mbOrgs even when co-cultured with healthy neurons.

As noted, our use of the mbOrgs revealed a compelling set of FTD-related phenotypes associated with TDP-43 LoF. This finding gave us confidence that the significant crSTMN2 increase in the 2D co-cultures of neurons and astrocytes was caused by the lack of expression of *GRN*. Thus, we tested the ability of recombinant PGRN to rescue this deficit in the context of *GRN*^−/−^ neurons and astrocytes. Our results show a striking ability to rescue crSTMN2 increases in the 2D culture, consistent with this observation being a direct result of loss of *GRN* and subsequent loss of PGRN function. It is important to note that, although treatment with PGRN can rescue crSTMN2 in the 2D co-culture, we did not find that treatment with PGRN was sufficient to rescue the observed phagocytosis deficits in the *GRN*^−/−^ iA. This might be because these astrocytes had been matured for 6–9 months before analysis, so the changes associated with loss of *GRN* may not be acutely rescuable. We believe that the multifunctional PGRN protein is likely to affect numerous aspects of cellular function; some acutely and some chronically. Thus, some deficits may be readily rescued by replacing PGRN and some may not. This is consistent with the recent finding that acute treatment with an engineered PGRN protein rescued some phenotypes of *GRN* deficient microglia, but not all phenotypes associated with *GRN* loss (Logan et al., 2021).

Like all model systems, iPSC-derived models have limitations. In particular, they do not fully recapitulate all features of the complex human brain and they currently do not incorporate microglia or vascular networks, although this may be possible in the future (Blanchard et al., 2021; Kumar et al., 2017; Mantle and Lee, 2018). This paradigm (both in 2D and 3D) is, however, amenable to the addition of any further cell types to make it an even more complete modeling tool. Despite these limitations, this approach provides a simple and straightforward method to model neurodegeneration that can be readily incorporated into laboratories and expanded to study basic cellular interactions and mechanisms of disease, revealing cell autonomous and non-autonomous roles for disease genes in astrocytes and neurons.

## Experimental procedures

### Resource availability

#### Corresponding authors

Further resources and reagents inquiries should be directed to and will be fulfilled by the corresponding authors (erik.ullian@ucsf.edu (martina@synapticure.com)

#### Materials availability

For questions about materials and methods, please contact with the corresponding authors.

### Human iPSC stem cell lines

Isogenic human iPSC line WTC11 and GRN^−/−^ iPSC line were generated by Dr. Bruce R. Conklin, as previously described (Miyaoka et al., 2014). *GRN*^−/−^ iPSC and *GRN*^−/−^ NGN2 iPSC were engineered (Figure S2F) and provided by Dr. Michael E. Ward (NIH) as previously described (Wang et al., 2017). iPSCs were cultured and maintained in Essential 8 Medium (Gibco, A1517001) on 6-well cell culture plates (Olympus, 25-105) coated with Vitronectin (Gibco, A14700) in DPBS. iPSCs were dissociated and passaged using EDTA (Invitrogen, AM9260G) in DPBS. This work was approved by UCSF GESCR Committee.

### Cortical-like neuronal induction

Cortical-like iN were generated as previously described (Fernandopulle et al., 2018). Briefly, iPSCs (WTC11) were expanded, dissociated, and replated on 10 μg/mL Matrigel (Corning, 354234) coated plates. Cells were grown in specialized iNeuron induction media (DMEM-F12 + Glutamax; Gibco, 10565-018), N-2 supplement (Gibco, 17502-048), MEM-NEAA (Gibco, 11140-050) containing doxycycline (Sigma, D3072) for approximately 72 h, with media changed every approximately 24 h. Cells were then dissociated using Accutase (Gibco, A1110501) and frozen in media +10% DMSO (Sigma, D8418) at high density to maximize cell viability.

### Statistical analyses

Statistical analyses were done using Prism 9.0 (GraphPad). If normally distributed, two-tailed unpaired Student’s t test was performed. If normally distributed, a one-way ANOVA was performed. A p value of less than 0.05 was considered significant. If non-normally distributed, a Mann-Whitney U-test was performed. A p value of less than 0.05 was considered significant. Using DESeq2, Wald tests were performed to evaluate genes for differential expression between conditions. The false discovery rate multiple testing correction method was then used for adjusted p values.

## Author contributions

M.dM., M.Koontz, and E.M.U. designed experiments. M.dM., M.Koontz, E.M., N.S., A.R., Y.K., S.L.G., N.M.D., and K.L. performed experiments and/or analyzed data. Y.M. generated the GRN^−/−^ iPSC lines. M.dM, E.M.U., E.M., H.L., and K.S. conceived the hypothesis. E.M., J.R.K, M.Kurnellas, M.Kampmann, M.E.W., E.J.H., and E.M.U. provided resources. M.dM., M.Koontz, E.M., and E.M.U. wrote the manuscript. All authors reviewed and approved the manuscript.

## Data Availability

Analyzed RNA-seq data will be made available upon request. The accession number for the fastq files data reported in this paper is https://www.ncbi.nlm.nih.gov/sra/PRJNA925944: SRA number PRJNA925944.

## Supplemental information

Document S1. Supplemental experimental procedures and Figures S1 and S2

Document S2. Article plus supplemental information
