# Supplementary material for: Granulin loss of function in human mature brain organoids implicates astrocytes in TDP-43 pathology
Source: Stem Cell Reports. 2023 Feb 23;18(3):706–19. doi: 10.1016/j.stemcr.2023.01.012 (PMC10031303; doi:10.1016/j.stemcr.2023.01.012)
Supplement: Document S1. Supplemental experimental procedures and Figures S1 and S2 [file mmc1.pdf]

Stem Cell Reports, Volume *18*

Supplemental Information

Granulin loss of function in human mature brain organoids implicates astrocytes in TDP-43 pathology

Martina de Majo, Mark Koontz, Elise Marsan, Nir Salinas, Arren Ramsey, Yien-Ming Kuo, Kyounghee Seo, Huinan Li, Nina Dräger, Kun Leng, Santiago L. Gonzales, Michael Kurnellas, Yuichiro Miyaoka, Joseph R. Klim, Martin Kampmann, Michael E. Ward, Eric J. Huang, and Erik M. Ullian

Supplementary Material

2D and 3D cell culture

iNeurons Validation: iNeurons were matured in 2D monoculture for four weeks by thawing and plating previously induced, harvested, and frozen iNeurons onto PDL (Sigma, P6407-5mg) coated six-well plates. iNeuron cultures were fed with BrainPhys Complete media (BrainPhys basal (StemCell Tech, 05790), Laminin (Gibco, 23017-015), NT3 (Peprotech, 450-03), BDNF (Peprotech, 450-02), N2 (Gibco, 17502-048), B27 -Vit.A (Gibco, 12587-010), HEPES (Gibco, 15630-106)) and maintained with ~2mls/well and a 50% media change every 72 hrs.

iAstrocyte validation: On approximately day 120, cultures were initially validated as astrocyte progenitor cells using ICC to confirm post-mitotic behavior, stellate morphology and canonical astrocyte gene expression (**FigS2 A-C**). On day 0 of ICC validation, a small number of spheroids were triturated into a single cell suspension and plated on Matrigel coated glass cover-slips at ~10k cells/cm^2 using ASM plus CNTF and BMP4 (Peprotech, 450-13 & 120-05ET). Media was changed every 48 hours. On day 7, cultures were fixed with 4% PFA and processed for ICC validation.

2D co-cultures: iNeurons and iAstrocytes were plated together at 1:1 ratio on Matrigel coated 24 well plates at a collective density of ~1x10^6 cells/well. Cells were maintained with BrainPhys Complete media at ~1ml/well with a 50% media change every 72 hrs.

3D co-cultures: iNeurons and iAstrocytes were maintained, validated, and cryo-preserved in order to maximize consistency and repeatability across multiple iterations of various experiments described herein. 3D mbOrgs co-cultures were prepared by thawing or dissociating constitutive cell types in single cell suspension. Cell types were then combined at specified ratios (1:1, iAst:iN) prior to being aliquoted into round-bottom plates. Plates were briefly centrifuged and left undisturbed for ~24 hours, allowing cells to coalesce into self-assembled spheroids. mbOrgs were maintained with a partial media change every 72 hours using BrainPhys Complete media.

Cortical-like astrocyte induction

Cortical-like iAstrocytes (iA) were generated as previously described (Krencik & Zhang, 2011). Human induced pluripotent stem cells (WTC11) were grown on vitronectin coated tissue culture plates using Essential 8 media. On day 0 of differentiation, iPSCs were dissociated into small aggregates averaging 50µm in diameter and transferred untreated tissue culture flasks with Neurosphere Induction Media (NSIM) (DMEM-F12/Neurobasal-A at 1:1 (Gibco, 10565-018: 108888-022)), N2 Supplement (Gibco, 17502-048), B27 -Vit.A Supplement (Gibco, 12587-010), MEM-NEAA (Gibco, 11140-050) plus SMAD inhibitors SB431542 (Stemcell Tech, 72234) and DMH1 (Tocris, 73634). Neurosphere induction media (NIM) plus SMAD inhibitors were changed every 48 hours. On day 7, once embryoid bodies began to show rosette clusters indicating early neuroepithelia morphological hallmarks, spheroids were transferred to Matrigel (Corning, 354230) coated tissue culture plates with NIM and SMAD inhibitors were removed. Media was changed every 24 hours until spheroids had sufficiently attached, and each spheroid exposed the rosette clusters within. On day 14, rosette clusters were mechanically removed and transferred to tissue culture flasks with NIM plus FGFb (Peprotech, 100-18B). Media was changed every 72 hours. On day 20, spheroids were triturated into a single cell suspension and transferred to a new untreated cell culture flask with astrocyte media (ASM) (DMEM-F12 (Gibco, 10565-018), N2 Supplement (Gibco, 17502-048), B27 -Vit.A Supplement (Gibco, 12587-010), Heparin (Stemcell Tech, 07980)) plus Y27632 (Tocris, 1254). From Day 28 to 180, spheroid aggregates were maintained in suspension with ASM plus EGF and FGFb (Peprotech, 100-15 & 100-18B) with media changes every 4-5 days. Spheroid aggregates were triturated every 7-10 days and transferred to new untreated tissue culture flasks.

Rescue experiment
Cells were plated as described above including an extra set of *GRN*$^{-/-}$ co-cultures which were treated with PGRN at 1µg/ml (Adipogen, AG-40A-0188Y). Media was partially changed and treatment replenished every 72 hours. Cells were then processed for RNA extraction or immunocytochemistry as described below.

Western blot
mbOrg or 2D cultures were lysed in RIPA buffer (Thermo Fisher Scientific Cat#PI89900) complemented with Phosphatase and Protease inhibitors (Thermo Fisher Scientific Cat#1862495 and 1862209) according to manufacturer's indications. Lysates were sonicated (QSonica, 55W, 110V Cat#Q55-110) for 10s/sample on ice and protein concentration was determined using Pierce BCA protein assay kit following manufacturer's instruction (Thermo Fisher Scientific cat#23227). 20µg to 30 µg of total protein from each lysate was combined with loading buffer (4X, Thermo Fisher Scientific, Cat#84788) and beta mercaptoethanol (0.05 M, BioRad Laboratories Cat#1610710) and loaded into a NuPAGE 4%–12% or 10% Bis-Tris Gel (Invitrogen, Cat# NP0336BOX, NP0301BOX) alongside a protein ladder (Thermo Fisher Scientific Cat#26619) to determine protein size. The gels were run in MOPS (Thermo Fisher Scientific Cat# NP0001) or MES (Invitrogen Cat#NP0002) buffer depending on the desired protein separation. Subsequently, the gel was transferred onto a nitrocellulose membrane using Trans Blot Sd Semi-Dry Transfer Cell (BioRad Cat#1703940) according to the manufacturer's instructions. After transfer, the membrane was stained with PonceauS, washed and blocked in 5% milk in PBS-T (PBS with 0.02% tween) or 10% BSA TBST-T (TBS + 0.02 tween), followed by overnight incubation with primary antibodies in 1% Milk or BSA at 4C (Table1). After incubation, the membrane was washed three times with PBS-T or TBS-T and then incubated with HRP conjugated secondary antibodies (Thermo Scientific Pierce Goat anti rabbit Cat#32260 or Goat anti mouse Cat#32230) at room temperature for 1hr. The membrane was then washed 3 times with PBS-T, incubated for two minutes with Pierce ECL western blotting substrate (Thermo Fisher Cat# 32106) and imaged at ChemiDoc (BioRad). Digital images were processed and analyzed using the image analysis software, ImageLab (BioRad).

Table 1. Primary antibodies used for western blot

Target	Vendor and Cat#	Concentration
GAPDH	Sigma-Aldrich, Cat# G8795	1:3000
PGRN	ThermoFisher, cat# 40-3400	1:250
TDP-43	Proteintech Cat#66734-1-1g	1:500
Phospho-TDP43 (Ser409/410)	Proteintech Cat#80007-1-RR	1:500
SYN1	Synaptic Systems Cat#101-004	1:300
PSD95	Abcam Cat#Ab18258	1:500

3D Organoid sample processing and cryosectioning for ICC
Samples were fixed in 4% PFA for ~25 minutes and washed three times with DPBS. Samples were then incubated overnight at 4°C in DPBS plus 30% sucrose and transitioned to a 1:1 solution of OCT & DPBS containing a final concentration of 30% sucrose. Samples were then incubated in OCT at RT for ~15 minutes prior to being embedded in OCT and frozen. OCT embedded samples were then cryosectioned at 20µm intervals using a Leica cryostat prior to applying the ICC protocol detailed below.

Immunocytochemistry

For 2D cultures, cells were gently washed in DPBS and fixed in 4% PFA (EMS; 50-980-487). Samples were washed three times in DPBS and incubated with blocking buffer (Glycine 0.1M, 5% goat/donkey serum, 1% BSA, 0.25% TritonX, 100mM glycine in DPBS) for one hour at room temperature. Samples were then incubated overnight with primary antibody (Table 2) at the appropriate concentration in primary blocking buffer (5% goat/donkey serum, 1%BSA, 0.25% triton X-100, 100nM glycine in DPBS) at 4C overnight, washed three times in DPBS and incubated with secondary antibodies (Table 3) diluted in secondary blocking buffer (10% BSA in DPBS) at room temperature for 1h. Samples were washed three times in DPBS and incubated with DAPI (Sigma-Aldrich Inc Cat#D9542-5MG) for 1 min at RT and washed twice in DPBS. If on coverslips, samples were mounted on microscope slides (Fisher Scientific Cat#1255015) using ProLong Gold (Invitrogen Cat#P36934) and dried at room temperature overnight. Sections were imaged at LSM900 confocal microscope (Zeiss) or Fluorescent microscopy images were acquired using a Revolve microscope (Echo Laboratories, San Diego, CA, USA). Images were analyzed using ImageJ(FIJI) (Schindelin et al., 2012).

For 3D cultures, slices were stained as described above. Briefly, we used a hydrophobic pen to delimitate the area of staining. We then washed with PBS to clean the excess OCT and start with the protocol described above. The PGRN antibody staining protocol included an antigen retrieval treatment by incubating tissue sections in 10 mM sodium citrate (pH 6.0) at 90°C for 10 minutes before the incubation in the blocking solution. For all 3D slices, labeling was done at room temperature, but samples were treated otherwise the same as 2D cultures.

Table 2. Primary antibodies used for ICC

Target	Vendor and Cat#	Concentration
NFIA	Sigma Cat#HPA008884	1:200 (2D) 1:400 (3D)
TUJ1	Millipore Cat#AB9354	1:200
AQP4	Sigma Cat#HPA014784	1:500
CD44	BD bioscience Cat#559046	1:200
GFAP δ	Milipore Cat#AB9598	1:500
TDP-43	Proteintech Cat#66734-1-1g	1:500
Phospho-TDP43 (Ser409/410)	Proteintech Cat#22309-1-AP	1:500
PGRN	R&D Sys. Cat#AF2557	1:100
PGRN	R&D Sys. Cat#AF2420	1:2000
MAP2	Abcam Cat#ab5392	1:500
PSD 95	Abcam Cat#ab13552	1:200
SYP	Synaptic Sys. Cat#101004	1:200

Table 3. Secondary antibodies used for ICC

Target	Vendor and Cat#	Concentration
Goat-Anti-Mouse Alexa 568	Thermofisher Scientific, A11004	1:500

Goat-Anti-Chicken Alexa 647	Thermofisher Scientific, A21449	
Goat-Anti-Rabbit Alexa 488	Thermofisher Scientific, A11008	
Donkey-Anti-Mouse Alexa 647	Thermofisher Scientific, A21202	
Donkey-Anti-Goat Alexa 594	Thermofisher Scientific, A11058	

IMARIS reconstruction
IMARISx64 was used to create a 3D reconstruction of the original z-stack using the different color channels.

Extranuclear TDP-43 analysis
TDP-43 quantifications were performed on FIJI. The DAPI channel and TDP-43 channel were each thresholded. Then, the image calculator was used to subtract the DAPI channel from the TDP-43 channel. Analyze Particles (0 μm^2 – infinity) was used on the resulting image to quantify the number and area of the non-nuclear TDP-43. The DAPI channel was then used to create a selection that was restored on the TDP-43 channel, such that the nuclear TDP-43 was highlighted, and the intensity could be measured in that region.

RNA isolation for bulk RNAseq and RT-PCR
RNA extraction was performed using the Trizol/phenol-Chloroform method (Sigma, T9424) as previously described (Krencik et al., 2015) and according to manufacturer specifications. Each sample contained ~50 mbOrgs, totaling ~2.5 $x10^6$ of cells per sample. The extracted RNA was used as a template for the synthesis of complementary DNA (cDNA) through reverse transcription, using iScript(tm) cDNA Synthesis Kit (Bio-Rad Cat#1708891) according to the manufacturer's protocol.

Quantitative PCR
cDNA samples were treated for genomic DNA contamination using TURBO DNA-free kit (Life Tech Cat#AM1907) per manufacturer's instructions. The cDNA was then diluted to a concentration of 5 ng/μl and 4 μl of each sample (total of 20 ng) were aliquoted in a white walled 96 well plate (Thermo Scientific) in technical duplicates. Samples were processed using PrimeTime std qPCR Assay (IDT) and IDT commercial standard conjugated primers for each of the genes analyzed except for cryptic STMN2 for which primers were designed by Kevin Eggan's lab and kindly shared with us (Forward: CTCAGTGCCTTATTCAGTCTTCTC, Probe: TCAGCGTCTGCACATCCCTACAAT, Reverse: TCTTCTGCCGAGTCCCATT). The quantitative PCR was run on Bio-Rad C1000 Thermal Cycler/CF96 Real-Time System following manufacturer's instructions. Data analysis was carried out applying the Pfaffl mathematical model for relative transcript quantification (Pfaffl, 2001) using RNA18S as a housekeeping gene.

RNA sequencing and analysis
RNA integrity was analyzed on an Agilent 2100 Bioanalyzer using RNA 6000 Nano kit (Agilent, 5067-1511). Only samples with an RNA integrity number (RIN) ≥ 9.4 were used to perform bulk RNA sequencing. Nugen Universal Plus (Tecan) was used as a library kit and libraries were sequenced on a SP300 flow cell of the Illumina NovaSeq 6000 machine with a paired-end 150 bp sequencing strategy (average depth 90 million reads/sample) at UCSF Genomics Core Facility. Genome was aligned to Ensembl Human.GRCh38.103. Kallisto 0.46.01 was used to generate transcript abundance files for each sample. Transcript counts files for each sample were generated using txImport and transcript differential analysis was performed using DESeq2 v1.24.0. A total of 6 samples were spread across two conditions.

Phagocytosis assay

Astrospheres were dissociated and counted. 10 wells at ~50k cells/well were plated in a 96 well/plate (~500k samples per condition). Astrocyte maturation was performed as previously described in iAstrocyte validation. One set of $GRN^{-/-}$ astrocytes was treated with PGRN 1 μg/ml (Adipogen, AG-40A-0188Y) every 72 hours. On the day of analysis, a subset of samples per condition were pre-treated with Cytochalasin D 10μM (Sigma #C2618) for 15 minutes to inhibit actin polymerization. Phagocytosis assay was performed as previously described (Dräger et al., 2022). Briefly, all samples were incubated with pHRodoRed-labeled synaptosomes at a concentration of 1 mg/ml in astrocyte media. Synaptosomes were isolated from fresh Innovative Grade US Origin Rat Sprague Dawley Brain (Innovative Research, Inc.; Cat. No. IGRTSDBR) with the Syn-PER™ Synaptic Protein Extraction Reagent (Thermo Scientific™; Cat. No. 87793) according to the manufacturer's protocol and then labelled with pHrodo™ Red, succinimidyl ester (pHrodo™ Red, SE) (ThermoFisher Scientific; Cat. No. P36600) as previously described (Dräger et al., 2022). Cells were washed twice with DPBS, dissociated, resuspended in ice-cold DPBS, and analyzed via flow cytometry. Flow cytometry data were analyzed using FlowJo (https://www.flowjo.com/).

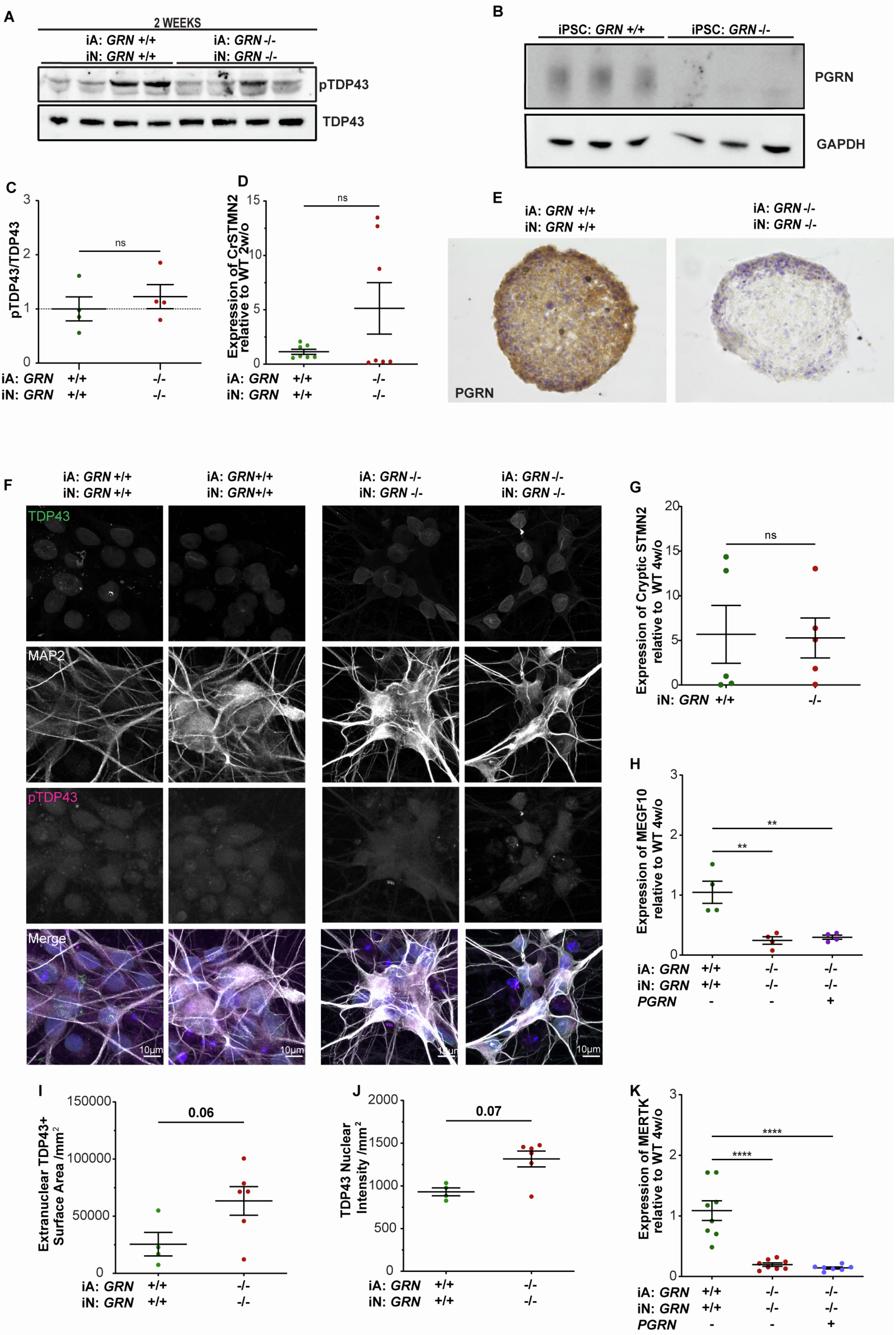

A
2 WEEKS
iA: GRN +/+
iN: GRN +/+
iA: GRN -/-
iN: GRN -/-
pTDP43
TDP43
B
iPSC: GRN +/+
iPSC: GRN -/-
PGRN
GAPDH
C
pTDP43/TDP43
ns
iA: GRN +/+ -/-
iN: GRN +/+ -/-
D
Expression of CrSTMN2 relative to WT 2w/o
ns
iA: GRN +/+ -/-
iN: GRN +/+ -/-
E
iA: GRN +/+
iN: GRN +/+
iA: GRN -/-
iN: GRN -/-
PGRN
F
iA: GRN +/+
iN: GRN +/+
iA: GRN+/+
iN: GRN+/+
iA: GRN -/-
iN: GRN -/-
iA: GRN -/-
iN: GRN -/-
TDP43
MAP2
pTDP43
Merge
10µm
G
Expression of Cryptic STMN2 relative to WT 4w/o
ns
iN: GRN +/+ -/-
H
Expression of MEGF10 relative to WT 4w/o
**
**
iA: GRN +/+ -/- -/-
iN: GRN +/+ -/- -/-
PGRN - - +
I
Extranuclear TDP43+ Surface Area /mm2
0.06
iA: GRN +/+ -/-
iN: GRN +/+ -/-
J
TDP43 Nuclear Intensity /mm2
0.07
iA: GRN +/+ -/-
iN: GRN +/+ -/-
K
Expression of MERTK relative to WT 4w/o
****
****
iA: GRN +/+ -/- -/-
iN: GRN +/+ -/- -/-
PGRN - - +

Supplementary figure 1. A) Western blot of mbOrg whole lysate showing similar expression on pTDP-43 in *GRN*$^{-/-}$ and *GRN*$^{+/+}$ mbOrg when normalized to total TDP-43 at two-week timepoint. C) Western blot quantification showing no difference in expression on pTDP-43 in *GRN*$^{-/-}$ compared to *GRN*$^{+/+}$ mbOrgs when normalized to total TDP-43 (n=4, unpaired t test, two tailed, p=ns, each n represents ~50 mbOrgs and was repeated independently four times). B) Western blot of WTC11 iPSCs whole lysate confirming GRN knock out in GRN$^{-/-}$ WTC11 lines. D) Quantification of CrSTMN2 expression using qPCR showing no difference in CrSTMN2 expression in *GRN*$^{-/-}$ compared to *GRN*$^{+/+}$ mbOrg after two weeks in culture (n=7±, unpaired t test, two tailed, p=<0.001, each n represents ~50 mbOrgs and the experiment was repeated independently thee times). E) Brightfield images of PGRN immunostaining in 3D cultures at 2-week timepoint showing the loss of PGRN in GRN$^{-/-}$ pure combination compared to *GRN*$^{+/+}$ pure combination. F) Representative ICC images of 2D neuronal cultures after for weeks. Cells were stained for TDP-43, MAP2 and pTDP-43 (scale bar 10µm). G) Quantification of CrSTMN2 expression using qPCR showing no difference in CrSTMN2 expression in GRN$^{-/-}$ compared to *GRN*$^{+/+}$ 2D neuronal cultures after four weeks in culture (n=5±, unpaired t test, one way ANOVA followed by multiple comparison, ns p>0.05, each n represents ~1x10^{6} cells and was repeated independently three times). H and K) Quantification of MERTK and MEGF10 expression using qPCR showing significantly MERTK and MEGF10 lower expression in *GRN*$^{-/-}$ compared to *GRN*$^{+/+}$ 2D cocultures after four weeks in culture. MERTK and MEGF10 expression is not rescued when *GRN*$^{-/-}$ co-cultures were treated with exogenous PGRN(n=4 or n=9 respectively, one way ANOVA followed by multiple comparison, **p<0.005, ****p>0.00005, each n represents ~1x10^{6} cells and was repeated independently three times). I and J) quantification of extranuclear TDP-43 per mm^{2} (I) and TDP-43 nuclear intensity per mm^{2} (J) in *GRN*$^{-/-}$ compared to *GRN*$^{+/+}$ mbOrg. Each dot represents one independent mbOrg (*GRN*$^{+/+}$ n=4, *GRN*$^{-/-}$ n=6, unpaired t test, two tailed, p<0.05, each n is one mbOrg, and the experiment was repeated independently 3 times). For all graphs data are represented as mean ± SEM.

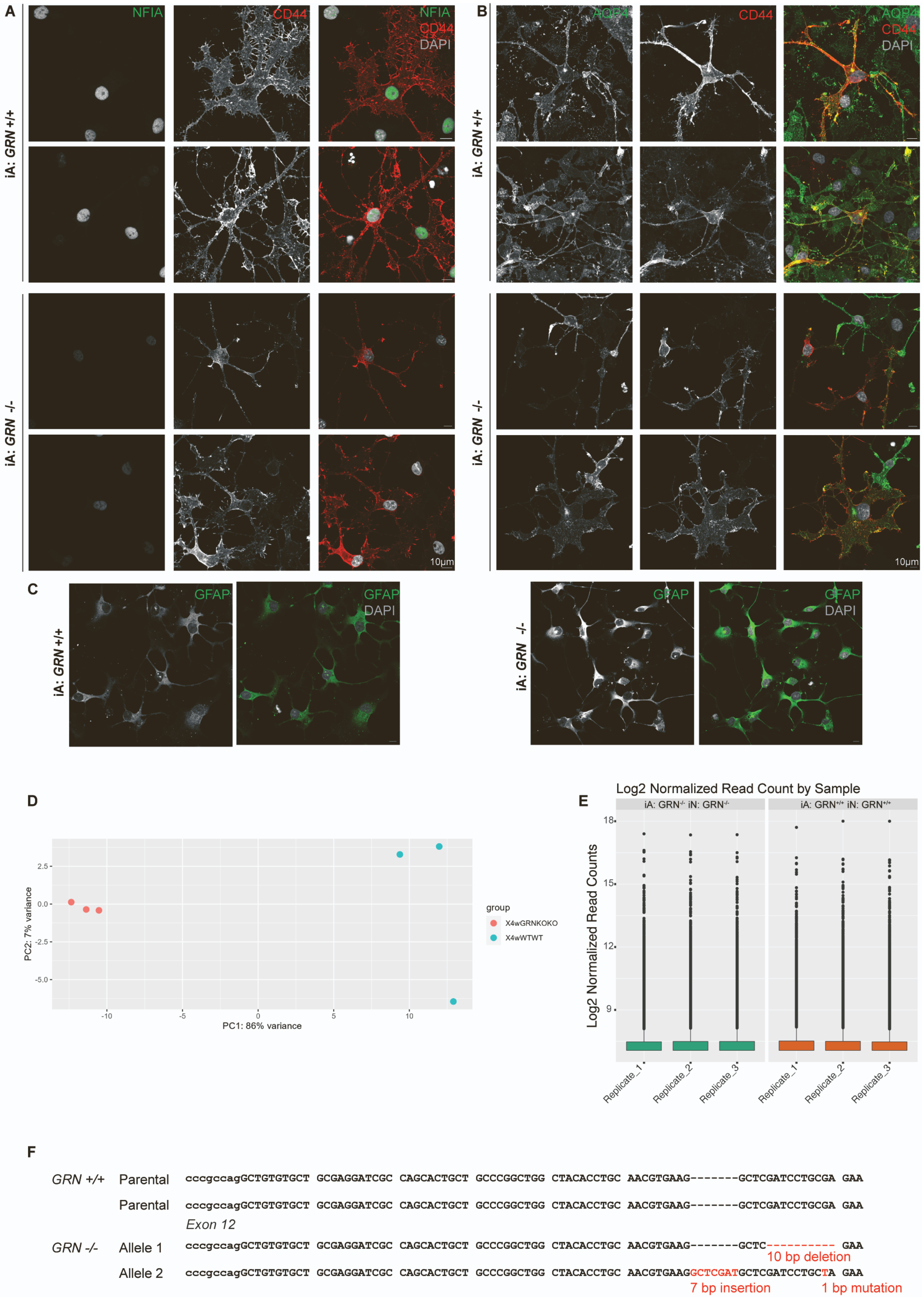
A
NFIA
CD44
NFIA
CD44
DAPI
iA: GRN +/+
iA: GRN -/-
10µm
B
AQP4
CD44
AQP4
CD44
DAPI
iA: GRN +/+
iA: GRN -/-
10µm
C
GFAP
GFAP
DAPI
iA: GRN +/+
iA: GRN -/-
D
PC2: 7% variance
PC1: 86% variance
group
X4wGRNKOKO
X4wWTWT
E
Log2 Normalized Read Count by Sample
Log2 Normalized Read Counts
Replicate_1
Replicate_2
Replicate_3
F
GRN +/+ Parental cccgccagGCTGTGTGCT GCGAGGATCGC CAGCACTGCT GCCCGGCTGG CTACACCTGC AACGTGAAG-------GCTCGATCCTGCGA GAA
Parental cccgccagGCTGTGTGCT GCGAGGATCGC CAGCACTGCT GCCCGGCTGG CTACACCTGC AACGTGAAG-------GCTCGATCCTGCGA GAA
Exon 12
GRN -/- Allele 1 cccgccagGCTGTGTGCT GCGAGGATCGC CAGCACTGCT GCCCGGCTGG CTACACCTGC AACGTGAAG-------GCTC---------- GAA
10 bp deletion
Allele 2 cccgccagGCTGTGTGCT GCGAGGATCGC CAGCACTGCT GCCCGGCTGG CTACACCTGC AACGTGAAGGCTCGATGCTCGATCCTGCTA GAA
7 bp insertion
1 bp mutation

Supplementary figure 2. A) Representative ICC images of 2D iA cultures stained for NFIA, CD44 and DAPI (scale bar 10µm) after one week differentiation B) Representative ICC images of 2D iA cultures stained for AQP4, CD44 and DAPI (scale bar 10µm) after 1 week differentiation. C) Representative ICC images of 2D iA cultures stained for GFAP and DAPI (scale bar 10µm) after one week differentiation. D) Principal components plot indicating distance between samples with principal component 1 (PC1) vs principal component 2 (PC2) and highlighting samples separation by genotype. E) Boxplots by sample for normalized read count values for all six analyzed samples. F) Diagram showing the *GRN* region in the WTC11iPSC line that was modified in the *GRN*$^{-/-}$ WTC11 iPSC. The modification results in a 10 base pair deletion (allele 1) and a 7+1 base pair insertion (allele 2) in Exon 12. These nonsense variants lead to the formation of a premature stop codon and a transcript that is degraded by nonsense mediated decay resulting in a *GRN* knock out iPSC line. For all graphs data are represented as mean ± SEM.

References

Pfaffl, M. W. (2001). A new mathematical model for relative quantification in real-time RT-PCR. *Nucleic Acids Research*, *29*(9), e45. https://doi.org/10.1093/nar/29.9.e45

Schindelin, J., Arganda-Carreras, I., Frise, E., Kaynig, V., Longair, M., Pietzsch, T., Preibisch, S., Rueden, C., Saalfeld, S., Schmid, B., Tinevez, J.-Y., White, D. J., Hartenstein, V., Eliceiri, K., Tomancak, P., & Cardona, A. (2012). Fiji: an open-source platform for biological-image analysis. *Nature Methods*, *9*(7), 676–682. https://doi.org/10.1038/nmeth.2019
